# *eFG*: an electronic resource for *Fusarium graminearum*

**DOI:** 10.1093/database/bat042

**Published:** 2013-06-22

**Authors:** Xiaoping Liu, Xiaodong Zhang, Wei-Hua Tang, Luonan Chen, Xing-Ming Zhao

**Affiliations:** ^1^Department of Computer Science, School of Electronics and Information Engineering, Tongji University, Shanghai 201804, China, ^2^Key Laboratory of Systems Biology, SIBS-Novo Nordisk Translational Research Centre for PreDiabetes, Shanghai Institutes for Biological Sciences, Chinese Academy of Sciences, Shanghai 200032, China, ^3^Institute of Systems Biology, Shanghai University, Shanghai 200444, China, ^4^National Key Laboratory of Plant Molecular Genetics, Institute of Plant Physiology and Ecology, Chinese Academy of Sciences, Shanghai, China, ^5^Collaborative Research Center for Innovative Mathematical Modelling, Institute of Industrial Science, University of Tokyo, Tokyo 153-8505, Japan

## Abstract

*Fusarium graminearum* is a plant pathogen, which causes crop diseases and further leads to huge economic damage worldwide in past decades. Recently, the accumulation of different types of molecular data provides insights into the pathogenic mechanism of *F. graminearum*, and might help develop efficient strategies to combat this destructive fungus. Unfortunately, most available molecular data related to *F. graminearum* are distributed in various media, where each single source only provides limited information on the complex biological systems of the fungus. In this work, we present a comprehensive database, namely *eFG* (**E**lectronic resource for ***F****usarium ****g****raminearum*), to the community for further understanding this destructive pathogen. In particular, a large amount of functional genomics data generated by our group is deposited in *eFG*, including protein subcellular localizations, protein–protein interactions and orthologous genes in other model organisms. This valuable knowledge can not only help to disclose the molecular underpinnings of pathogenesis of the destructive fungus *F. graminearum* but also help the community to develop efficient strategies to combat this pathogen. To our best knowledge, *eFG* is the most comprehensive functional genomics database for *F. graminearum* until now. The *eFG* database is freely accessible at http://csb.shu.edu.cn/efg/ with a user-friendly and interactive interface, and all data can be downloaded freely.

**Database URL:** http://csb.shu.edu.cn/efg/

## Introduction

The filamentous ascomycete *Fusarium graminearum* (teleomorph *Gibberella zeae*) is the major pathogenic agent of *Fusarium* head blight (FHB) and *Fusarium* ear rot (1), which can cause diseases for wheat, barley, maize and other crops, leading to yield loss and food quality problems, and are becoming serious problems in many countries over the world. In general, FHB causes diseases to crops within a few weeks, and results in huge economic loss (2). Most importantly, this pathogen produces some mycotoxins, e.g. deoxynivalenol and zearelanone, which contaminate food products and therefore increase health risks (3, 4). However, it is difficult to fight this destructive fungus whose pathogenic mechanism is known to a limited extent (5, 6).

Recently, the accumulation of different kinds of molecular data provides invaluable information on the biology of *F. graminearum*, which can help to develop effective strategies to fight this fungus. For example, the complete genome of *F. graminearum* provides insights into the possible genome regions enriched for infection-related genes (7). A comprehensive genome database FGDB (*Fusarium graminearum* Genome Database) provides information on manually revised gene set (8). On the other hand, some ‘omics’ data provide valuable information on the biological systems inside the fungus. For example, our recently predicted protein–protein interactions deposited in FPPI database (9) give a global interactome map of *F. graminearum* proteins; gene expression data from PLEXdb database (http://www.plexdb.org/) (10) describes the transcriptional activity under distinct conditions; pathway information available in KEGG database (11) characterizes the context in which genes function.

Unfortunately, most of the valuable information described above is distributed in various ways: some are deposited in public databases while some are just described in literature, where each single source can only provide limited information on the complex biological systems of the fungus *F. graminearum.* Therefore, it is necessary to construct a ready-to-use comprehensive molecular database for *F. graminearum*. To fulfill this gap, in this work, we build such a uniform database, namely *eFG* (**E**lectronic resource for ***F****usarium ****g****raminearum*), which contains both genome and systematic functional information for *F. graminearum*. Compared with existing databases for *Fusarium* genus, e.g. CiF (http://www.fusariumdb.org/), FungiDB (http://fungidb.org/fungidb/), CFGP (http://cfgp.riceblast.snu.ac.kr/) and EnsemblFungi (http://fungi.ensembl.org/), *eFG* database is more comprehensive and provides some novel and specific information for *F. graminearum*. In *eFG* database, except for genome information collected from public databases, we also incorporate some functional annotations, such as pathway annotation, enzyme families and transcription factors. In particular, *eFG* contains a protein interactome map, protein subcellular localization annotations, pathogenic genes and *F. graminearum* orthologous genes in other species (including fungi, bacteria and mammalian), all of which are predicted by our group in our previous works (9, 12, 13). These derived functional genomics data can help us to understand the possible functions of *F. graminearum* proteins. For example, the subcellular localization data gives a spatial cellular landscape of whole genome proteins within a cell, while the orthologous information can help to annotate unknown genes by transferring annotations between orthologs. As a case study, by integrating data deposited in *eFG* database, we show that the pathogenic genes of *F. graminearum* have different molecular characteristics compared with whole genome background, e.g. higher degree in the interactome map and enriched in MAPK signaling pathway and cysteine and methionine metabolism. We believe that the comprehensive database *eFG* can shed light on the molecular mechanisms underlying pathogenesis of *F. graminearum*, and help the community to develop efficient strategies to combat this pathogen. The database can be freely accessed through distinct browsers, including Internet Explorer (version 9/10), Firefox (version 15/16), Google Chrome and Safari (Version 6), where all the data can be freely downloaded for academic purpose.

## Database Construction

### Database overview

**Figure 1.**
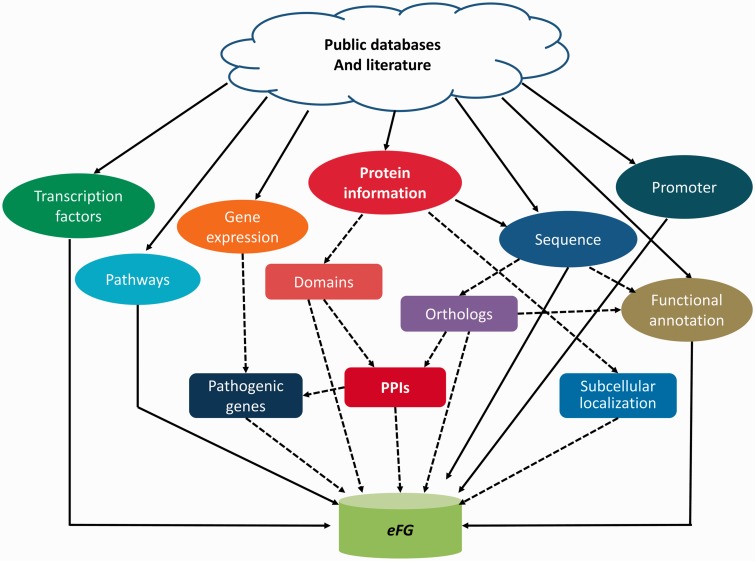
Schematic view of the *eFG* database, where ellipses denote data collected from public databases or literature while rectangles denote those derived data, and dashed lines represent the inference procedure.

The *eFG* database integrates different kinds of data, including genome information (gene and protein sequence, promoter sequence), proteome information (protein domain architecture, protein subcellular localization, protein–protein interaction) and functional annotations (pathogenic gene, transcription factor, catalytic activity of enzyme, pathway, gene ontology term and orthologs), into a uniform database (Figure 1). All the data deposited in *eFG* can be freely downloaded for academic use. Furthermore, *eFG* provides access to gene expression data measured under different conditions deposited in GEO (Gene Expression Omnibus) (14) and PLEXdb (15) databases for further analysis.

**Figure 2.**
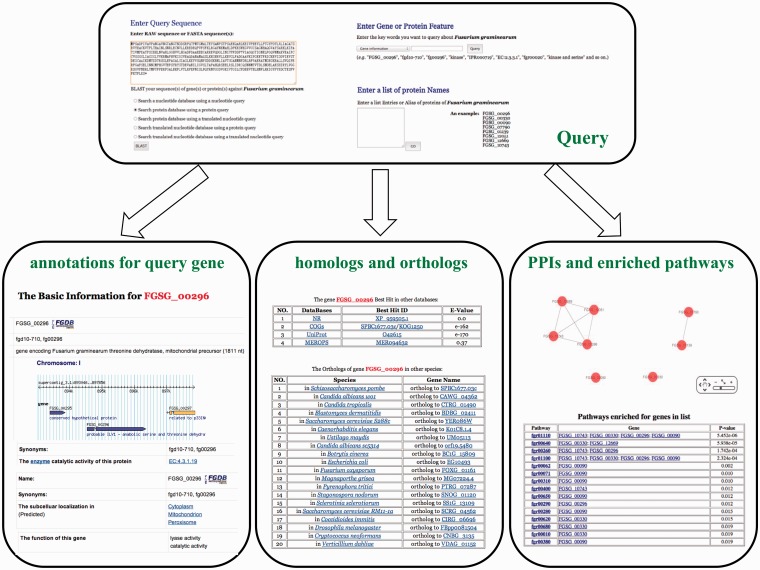
Schematic diagram of interactive querying interface of *eFG*. This figure shows the three basic query interface and parts of retrieved results including basic annotations, homologs, orthologs, PPI and enriched pathways.

In addition, a user-friendly interactive interface was constructed for querying genes of interest. By submitting gene symbols (e.g. FGSG_00296), one can retrieve annotations of interest, homologs in other databases, and orthologs in other species, among others, for this gene by selecting distinct drop-down options (Figure 2). Furthermore, one can also retrieve corresponding genes’ information by identifiers of enzymatic function [e.g. query with ‘EC:1.3.5.1’ can return genes with the catalytic function of ‘succinate dehydrogenase (ubiquinone)’], protein domains (e.g. query with ‘IPR001926’ can retrieve the genes which contain the domain of ‘Pyridoxal phosphate-dependent enzyme, beta subunit’), KEGG pathway (e.g. query with ‘fgr00260’ can present all genes which are included in the ‘glycine, serine and threonine metabolism’ pathway) and annotation key word (e.g. ‘kinase’ and ‘transferase’ can respectively return the genes that are annotated with the key words). In addition, logical combination by word ‘AND’ (e.g. key words ‘kinase and serine’ can list the genes which are kinases and contain serine) is also supported. One can retrieve all available information for a single gene, including sequence information, localization information, domain information, pathogenic information, TF (transcription factor) information, enzyme catalytic information, pathway information, protein–protein interactions, orthologs information and best hit homologs in other databases. Specifically, one can query an unknown sequence with BLAST (Basic Local Alignment Search Tool) (16) running in the background. Moreover, the *eFG* database allows querying a set of genes and retrieves comprehensive information on the gene set. With the batch input of a set of genes, one is able to investigate the functional relationships among these genes, e.g. protein–protein interaction or within the same pathway (Figure 2). For instance, the possible interactions between these gene products are firstly retrieved from the interactome map and are then shown in a graph visualized with Cytoscape Web (17), a web implementation of Cytoscape (18). It enables the user to view the network in an interactive way, such as panning and zooming in/out the network without changing the original layout, and dragging/clicking the nodes. Subsequently, pathways and GO (gene ontology) terms that are associated with queried proteins are listed with corresponding *P*-values calculated based on hypergeometric test to show those ones in which the queried proteins are enriched. In addition, one can query the *eFG* database by simply submitting gene sequence(s) if the gene(s) of interest is (are) not known, where the BLAST is run in the background to retrieve the best similar genes/proteins in the *F. graminearum* genome (Figure 2).

Beyond above characteristics, the *eFG* database provides cross-references to other databases. For example, one can link to KEGG database by clicking the retrieved pathways for the queried genes. Similarly, for the orthologs of one *F. graminearum* gene, one can link to the original databases against which the orthologs are recognized, where these databases provide more detailed information about those orthologs so that the function of the *F. graminearum* gene can be easily inferred.

### Database content

#### F. graminearum *genome*

The full genome of *F. graminearum* was finished in 2006 (19), which was manually revised later and deposited in the FGDB database (8). The assembled FG3 genome (version 3.1) that contains potential protein sequences and the function annotations for corresponding genes were downloaded from FGDB. These data were imported into the *eFG* database, which results in 13 719 genes with corresponding upstream 1000 base pairs sequence from its transcription start site for each gene, where the possible function annotations for these genes were organized in FunCat format (20). Moreover, protein domains were identified with InterProScan (21) for all potential proteins and were deposited into *eFG*.

#### Transcription Factors

**Figure 3.**
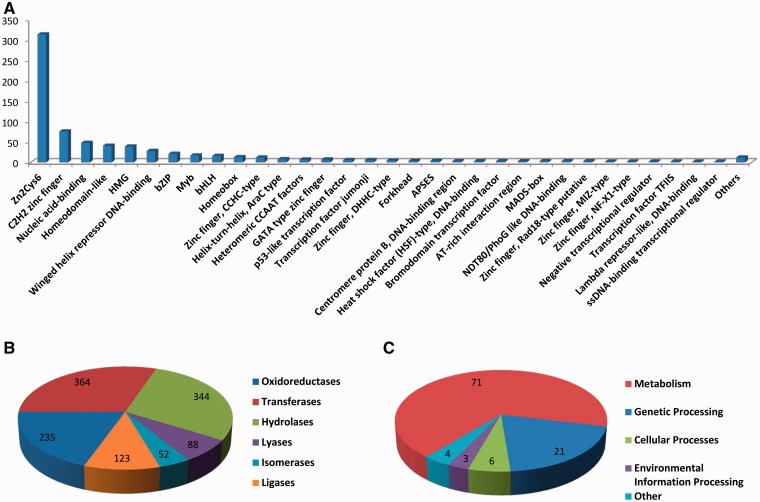
(**A**) The distribution of TFs in different TF families, (**B**) the distribution of the known enzyme functional groups, (**C**) the distribution of the known pathways.

The transcription factors (TFs) are important regulators that modulate transcriptional program, which is one of the most important biological processes. In *eFG* database, the TFs of *F. graminearum* were collected from published literature (22). Right now, there are in total 717 potential TFs belonging to 44 TF families (Figure 3A), where the Zn2Cys6 family is the biggest one containing fungus-specific transcriptional regulatory proteins with an N-terminal Cys-rich motif and plays essential roles in both primary and secondary metabolism, drug resistance and meiotic development (23).

#### *F. graminearum* Enzyme Proteins

The enzyme proteins are important to various biochemical reactions which are generally catalyzed by these proteins. Enzyme Commission number (EC number) is a numerical classification scheme for enzymes based on the biochemical reactions that they are involved in, and is used to identify the catalytic activities of *F. graminearum* enzyme proteins here. We collected 1206 enzyme proteins with known catalytic activities from KEGG database and imported them into the *eFG* database. As shown in Figure 3B, the two largest groups of enzyme proteins in *F. graminearum* are transferases and hydrolases.

#### *F. graminearum* pathways and GO information

In cells, most genes or their products participate in different pathways to exert their functions, including genetic pathways, metabolic pathways, and signal transduction pathways. These pathways play essential roles in development, cell fate, and even invading host, which can help to understand the mechanisms of fungal pathogenesis which in turn help to design effective strategies to combat the fungus. In *eFG* database, we collected 105 pathways in which 1374 *F. graminearum* proteins can be found from KEGG database. As shown in Figure 3C, among the 105 pathways, most of the *F. graminearum* proteins that have pathway annotations are located in metabolism pathways. The GO (gene ontology) database provides functional annotations for genes and their products across distinct species (24). To make *eFG* a more comprehensive database, the annotations for *F. graminearum* genes were obtained from EBI FTP site (ftp://ftp.ebi.ac.uk/pub/databases/GO/goa/proteomes/22027.G_zeae.goa) and imported into *eFG*. As a result, there are 4658 GO terms for cell component, 14 198 GO terms for molecular function and 8991 GO terms for biological process, that are annotated to *F. graminearum* genes.

#### Subcellular Localizations

Protein subcellular localization information describes the spatial arrangement of proteins within cells, thereby providing important functional information on proteins. However, it is a laborious and time consuming task to experimentally determine the subcellular localization of proteins. In our previous work, one computational approach based on Support Vector Machine (SVM) and protein primary structure (12) was proposed to predict the subcellular locations of *F. graminearum* proteins. In addition, for the *F. graminearum* proteins that have significant sequence similarity to those in a non-redundant dataset for fungi collected from UniProtKB database with subcellular localization annotation, sequence alignment was used to transfer annotations of homologous proteins to uncharacterized *F. graminearum* proteins so that the *F. graminearum* proteins are annotated more comprehensively. In *eFG* database, the predicted subcellular localizations of 12 786 proteins were clustered into 22 groups (Table 1).

**Table 1.** Distribution of the subcellular localizations for 12 786 *F. graminearum* proteins

Subcellular location	No.	Subcellular location	No.
Secreted	3163	Lipid-anchor	61
Cytoplasm	5699	Centromere	23
Endoplasmic reticulum	4166	Kinetochore	28
Golgi apparatus	1975	Telomere	19
Nucleus	2868	Cytoskeleton	88
Mitochondrion	4484	Spindle	48
Peroxisome	2315	Prospore membrane	4
Endosome	1114	Peripheral membrane	280
Vacuole	3505	Multi-pass membrane	968
Cell membrane	5130	Single-pass membrane	229
Vacuole membrane	203	Preautophagosomal structure membrane	4

#### *F. graminearum* Orthologs and Homologs

The orthologs of *F. graminearum* genes in other well-studied organisms can help to annotate uncharacterized *F. graminearum* genes. By using an existing tool, InParanoid (25), we identified the orthologs of *F. graminearum* genes in 24 organisms (Table 2), where the most evolutionally related species have the largest number of orthologs in *F. graminearum*. These orthologous information can help to understand the possible functions of *F. graminearum* genes.

**Table 2.** Numbers of *F. graminearum* orthologs in other organisms

Species	No.	Species	No.
*Caenorhabditis elegans*	1944	*Coccidioides posadasii*	5401
*Drosophila melanogaster*	2063	*Cryptococcus neoformans*	3281
*Escherichia coli*	558	*Fusarium oxysporum*	9419
*Homo sapiens*	2389	*Histoplasma capsulatum*	4718
*Mus musculus*	2383	*Magnaporthe grisea*	6065
*Schizosaccharomyces pombe*	2892	*Pyrenophora tritici*	6225
*Blastomyces dermatitidis*	5379	*Saccharomyces cerevisiae RM11-1a*	2728
*Botrytis cinerea*	5865	*Sclerotinia sclerotiorum*	5978
*Candida albicans sc5314*	3262	*Stagonospora nodorum*	6545
*Candida albicans wo1*	3155	*Ustilago maydis*	3166
*Candida tropicalis*	3133	*Verticillium dahliae*	6986
*Coccidioides immitis*	5405	*Saccharomyces cerevisiae S288c*	2753

In addition, the best-hit homologs in public databases were recognized for *F. graminearum* genes. In the *eFG* database, those genes that are most similar to each *F. graminearum* gene were picked from four widely used public databases, including non-redundant protein sequences database (NR, ftp://ftp.ncbi.nih.gov/blast/db/FASTA/) (26), universal protein resource database (UniProt, http://www.uniprot.org/) (27), clusters of orthologous groups of proteins (COGs, http://www.ncbi.nlm.nih.gov/COG/) (28) and MEROPS (http://merops.sanger.ac.uk/) (29). As a result, there are 12 922 genes from NR, 12 650 genes from COGs, 11 846 genes from UniProt and 11 612 genes from MEROPS, which are most similar to at least one *F. graminearum* gene.

#### Protein–protein Interactions

**Figure 4.**
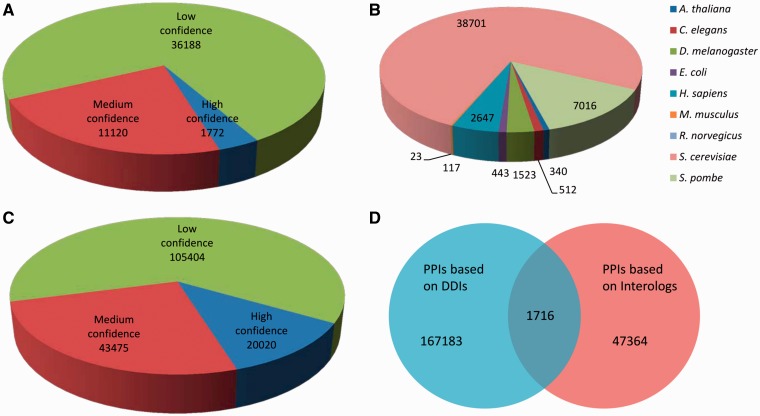
Distribution of protein–protein interactions. (**A**) Distribution of PPIs predicted from interologs-based method. (**B**) Number of PPIs inferred from different organisms (with possible overlaps). (**C**) Distribution of PPIs predicted based on DDIs. (**D**) Vienn diagram of interactions inferred from both interologs and DDIs.

Protein–protein interactions (PPIs) are important to biological functions (30). In our previous work, a computational framework was presented to predict PPIs for *F. graminearum* based on both interologs and domain–domain interactions (9, 31). Here, the interactome of *F. graminearum* was extended based on new datasets available. In the interologs method, two proteins are regarded as an interaction pair in *F. graminearum* if their corresponding orthologs in any other organism are known to interact with each other. Finally, 49 080 interactions were predicted based on *F. graminearum* orthologs from nine well-studied species, including *Arabidopsis thaliana*, *Caenorhabditis elegans*, *Drosophila melanogaster*, *Escherichia coli*, *Homo sapiens*, *Mus musculus*, *Rattus norvegicus*, *Saccharomyces cerevisiae* and *Schizosaccharomyces pombe* by using InParanoid. According to the confidence classification rules described in (32), these interactions can be classified into high-confidence, medium-confidence and low-confidence (Figure 4A). The numbers of interactions supported by each organism along with corresponding number of proteins are shown in Figure 4B. The underlying principle for the prediction of protein–protein interactions based on domain–domain interactions is that two proteins interact if and only if at least one pair of domains from the two proteins are known to interact. The domains within *F. graminearum* proteins were annotated by using PfamScan available from Pfam Web site (33). Finally, 168 899 interactions predicted from DDIs (Domain–domain interactions) were also classified into three confidence levels (Figure 4C) as described in (32).

In total, 216 263 interactions involving 6741 unique proteins were predicted, where 1716 interactions were predicted by both methods (Figure 4D). Furthermore, we constructed a core PPI dataset that contains high-confidence interactions predicted by either interologs or DDIs and those predicted by both methods but not necessarily to be highly confident. There are in total 34 675 interactions between 4047 proteins in the core set. All these protein interactions can be found in *eFG* database and freely downloadable from the Web site.

#### Pathogenic Genes

In *eFG* database, we also collected pathogenic genes for *F. graminearum* from literature. Moreover, the pathogenic genes predicted in our previous work (13) were also imported into *eFG* database. In brief, those genes that interact with known pathogenic genes are more likely to be pathogenic genes. With the core PPI dataset and known pathogenic genes from PHI-base database (http://www.phi-base.org/) (34) as seed genes, pathogenic modules were identified based on the genes differentially expressed before and after the invasion of *F. graminearum*, where the genes in the module were regarded as putative pathogenic genes. Right now, there are in total 100 pathogenic genes deposited in *eFG* database.

### Case study: characteristics of pathogenic genes

Understanding the molecular underpinning of *F. graminearum* pathogenesis is important for developing efficient strategies to combat this fungus. Therefore, using the information extracted from *eFG* database, we investigated whether there are specific molecular patterns associated with pathogenic genes of *F. graminearum*.

By submitting the 100 pathogenic genes to *eFG* database with multi-genes querying, we found that these genes are significantly enriched in two pathways: MAPK signaling pathway (*P*-value 1.91 × 10^−^^5^) and cysteine and methionine metabolism (*P*-value 1.64 × 10^−^^3^), which is consistent with previous findings that MAPK pathway is involved in the pathogenesis of phytopathogenic fungi (35). The enrichment of cysteine and methionine metabolism indicates that those known pathogenic genes of *F. graminearum* may participate in the synthesis of sulfur-containing amino acids.

The enzyme catalytic activity analysis indicates that 19 pathogenic genes are enzymes, among which 11 are transferases, implying that transferases are more important for *F. graminearum* to infect its host. Furthermore, there is one oxidoreductase, one isomerase, two hydrolases, two lyases and two ligases in the 19 pathogenic genes. With function annotations obtained from *eFG* for the pathogenic genes, we found that 29 pathogenic genes are kinase, 14 are synthase, 7 are cyclin-dependent kinases, and 6 are involved in MAPK pathway.

**Figure 5.**
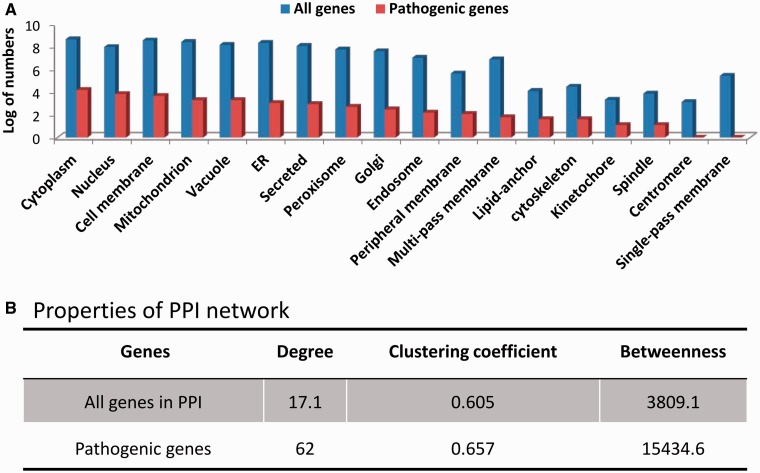
(**A**) Distribution of subcellular localizations for all genes and pathogenic genes. (**B**) Network parameters of pathogenic genes and background genes.

In addition, we investigated the subcellular localizations of pathogenic genes, which occur in 18 of 22 subcellular locations (Figure 5A).We found that the distribution of subcellular localizations of pathogenic genes is significantly (*P*-value of 2.63 × 10^−^^6^) different from that of the whole genome genes. The most frequent subcellular localizations in which pathogenic genes occur include cytoplasm, nucleus and cell membrane.

Investigating the pathogenic genes in the context of protein interactome, we found that these genes are significantly different from the whole-genome background with respect to three important network parameters, i.e. degree, clustering coefficient and betweenness (36, 37) (Figure 5B). The pathogenic genes are found to have higher degree and betweenness, which indicates that pathogenic genes tend to connect more genes, thereby playing important roles in the biological processes.

In summary, from above analysis, we can see that there are possible specific molecular patterns associated with pathogenic genes of *F. graminearum*, and these patterns can help to predict new potential pathogenic genes in the future.

## Conclusion

We presented a comprehensive database for *F. graminearum*, namely *eFG*, which integrates different kinds of molecular data from literature and inferred from existing data by our group into the uniform resource. Furthermore, an interactive powerful querying interface was also constructed to meet different requirements of biologists, from which biologists can get desired results by providing the key words that they are interested in. We believe that this valuable database can benefit the community not only for better understanding the pathogenic agent *F. graminearum*, but also for developing efficient strategies to combat this pathogen.

## Funding

National Natural Science Foundation of China (61103075, 91130032, 91029301, 61134013); Innovation Program of Shanghai Municipal Education Commission (13ZZ072); Shanghai Pujiang Program and JSPS/CSTP through the FIRST Program. Funding for open access charge: National Natural Science Foundation of China (91130032).

*Conflict of interest*. None declared.

## References

[1] Miedaner T, Cumagun CJR, Chakraborty S (2008). Population genetics of three important head blight pathogens *Fusarium graminearum*, F-pseudograminearum and F-culmorum. J. Phytopathol..

[2] McMullen M, Jones R, Gallenberg D (1997). Scab of wheat and barley: a re-emerging disease of devastating impact. Plant Dis..

[3] Pestka JJ, Smolinski AT (2005). Deoxynivalenol: toxicology and potential effects on humans. J. Toxicol. Environ. Health B Crit. Rev..

[4] Delserone LM (2007). *Fusarium* mycotoxins: chemistry, genetics, and biology. J. Agricult. Food Info..

[5] Walter S, Nicholson P, Doohan FM (2010). Action and reaction of host and pathogen during *Fusarium* head blight disease. New Phytol..

[6] Kazan K, Gardiner DM, Manners JM (2012). On the trail of a cereal killer: recent advances in *Fusarium graminearum* pathogenomics and host resistance. Mol Plant Pathol..

[7] Cuomo CA, Gueldener U, Xu JR (2007). The *Fusarium graminearum* genome reveals a link between localized polymorphism and pathogen specialization. Science.

[8] Wong P, Walter M, Lee W (2011). FGDB: revisiting the genome annotation of the plant pathogen *Fusarium graminearum*. Nucleic Acids Res..

[9] Zhao XM, Zhang XW, Tang WH (2009). FPPI: *Fusarium graminearum* protein-protein interaction database. J. Proteome Res..

[10] Wise RP, Caldo RA, Hong L (2007). BarleyBase/PLEXdb. Methods Mol. Biol..

[11] Kanehisa M (2002). The KEGG database. Novartis Found. Symp..

[12] Sun C, Zhao XM, Tang W (2010). FGsub: *Fusarium graminearum* protein subcellular localizations predicted from primary structures. BMC Syst. Biol..

[13] Liu X, Tang WH, Zhao XM (2010). A network approach to predict pathogenic genes for *Fusarium graminearum*. PLoS One.

[14] Edgar R, Domrachev M, Lash AE (2002). Gene expression omnibus: NCBI gene expression and hybridization array data repository. Nucleic Acids Res..

[15] Dash S, Van Hemert J, Hong L (2012). PLEXdb: gene expression resources for plants and plant pathogens. Nucleic Acids Res..

[16] Altschul SF, Gish W, Miller W (1990). Basic local alignment search tool. J. Mol. Biol..

[17] Lopes CT, Franz M, Kazi F (2010). Cytoscape Web: an interactive web-based network browser. Bioinformatics.

[18] Shannon P, Markiel A, Ozier O (2003). Cytoscape: a software environment for integrated models of biomolecular interaction networks. Genome Res..

[19] Guldener U, Mannhaupt G, Munsterkotter M (2006). FGDB: a comprehensive fungal genome resource on the plant pathogen *Fusarium graminearum*. Nucleic Acids Res..

[20] Ruepp A, Zollner A, Maier D (2004). The FunCat, a functional annotation scheme for systematic classification of proteins from whole genomes. Nucleic Acids Res..

[21] Quevillon E, Silventoinen V, Pillai S (2005). InterProScan: protein domains identifier. Nucleic Acids Res..

[22] Ma LJ, Van Der Does HC, Borkovich KA (2010). Comparative genomics reveals mobile pathogenicity chromosomes in *Fusarium*. Nature.

[23] Todd RB, Andrianopoulos A (1997). Evolution of a fungal regulatory gene family: the Zn(II)2Cys6 binuclear cluster DNA binding motif. Fungal Genet. Biol..

[24] Gene Ontology Consortium (2008). The gene ontology project in 2008. Nucleic Acids Res..

[25] Ostlund G, Schmitt T, Forslund K (2010). InParanoid 7: new algorithms and tools for eukaryotic orthology analysis. Nucleic Acids Res..

[26] Pruitt KD, Tatusova T, Maglott DR (2005). NCBI Reference Sequence (RefSeq): a curated non-redundant sequence database of genomes, transcripts and proteins. Nucleic Acids Res..

[27] Apweiler R, Bairoch A, Wu CH (2004). UniProt: the universal protein knowledgebase. Nucleic Acids Res..

[28] Tatusov RL, Koonin EV, Lipman DJ (1997). A genomic perspective on protein families. Science.

[29] Rawlings ND, Barrett AJ (1999). MEROPS: the peptidase database. Nucleic Acids Res..

[30] Zhao XM, Wang RS, Chen L (2008). Uncovering signal transduction networks from high-throughput data by integer linear programming. Nucleic Acids Res..

[31] Zhao XM, Chen L, Aihara K (2010). A discriminative approach for identifying domain-domain interactions from protein-protein interactions. Proteins.

[32] Sapkota A, Liu X, Zhao XM (2011). DIPOS: database of interacting proteins in oryza sativa. Mol. Biosyst..

[33] Finn RD, Mistry J, Tate J (2010). The Pfam protein families database. Nucleic Acids Res..

[34] Winnenburg R, Baldwin TK, Urban M (2006). PHI-base: a new database for pathogen host interactions. Nucleic Acids Res..

[35] Idnurm A, Howlett BJ (2001). Pathogenicity genes of phytopathogenic fungi. Mol. Plant Pathol..

[36] Costa LDF, Rodrigues FA, Travieso G (2007). Characterization of complex networks: a survey of measurements. Adv. Phys..

[37] Boccaletti S, Latora V, Moreno Y (2006). Complex networks: structure and dynamics. Phys. Rep..

